# Radar Emitter Signal Recognition Based on One-Dimensional Convolutional Neural Network with Attention Mechanism

**DOI:** 10.3390/s20216350

**Published:** 2020-11-07

**Authors:** Bin Wu, Shibo Yuan, Peng Li, Zehuan Jing, Shao Huang, Yaodong Zhao

**Affiliations:** 1 School of Electronic Engineering, Xidian University, Xi’an 710071, China; bwu@xidian.edu.cn (B.W.); li_peng001@163.com (P.L.); m18903513779@163.com (Z.J.); 2 Science and Technology on Electronic Information Control Laboratory, Chengdu 610036, China; huangshaolee@163.com (S.H.); zhyd0921@163.com (Y.Z.)

**Keywords:** radar emitter signal recognition, one-dimensional convolutional neural network, attention mechanism

## Abstract

As the real electromagnetic environment grows complex and the quantity of radar signals turns massive, traditional methods, which require a large amount of prior knowledge, are time-consuming and ineffective for radar emitter signal recognition. In recent years, convolutional neural network (CNN) has shown its superiority in recognition so that experts have applied it in radar signal recognition. However, in the field of radar emitter signal recognition, the data are usually one-dimensional (1-D), which takes more time and storage space than by using the original two-dimensional CNN model directly. Moreover, the features extracted from convolutional layers are redundant so that the recognition accuracy is low. In order to solve these problems, this paper proposes a novel one-dimensional convolutional neural network with an attention mechanism (CNN-1D-AM) to extract more discriminative features and recognize the radar emitter signals. In this method, features of the given 1-D signal sequences are extracted directly by the 1-D convolutional layers and are weighted in accordance with their importance to recognition by the attention unit. The experiments based on seven different radar emitter signals indicate that the proposed CNN-1D-AM has the advantages of high accuracy and superior performance in radar emitter signal recognition.

## 1. Introduction

Radar emitter signal recognition is a technology used to obtain information about radar systems by intercepting and analyzing their signals. The features of radar signals are always extracted manually based on traditional methods. Much research has been done on feature extraction. Bouchou et al. [1] calculated eight key features, including higher-order cumulants (HOC), and used stacked sparse autoencoder (SSAE) to recognize seven different digital modulation signals. Park et al. [2] used wavelet features and support vector machines (SVM) to recognize eight different digital modulation signals. However, as the real electromagnetic environment grows complex and the quantity of radar signals turns massive, the performance of traditional methods, which require a great deal of prior knowledge and time, is poor when the radar emitter signals are on low signal-to-noise ratio (SNR).

It is expected to develop a generic and effective method that can automatically extract features from radar signals. Deep learning [3] has attracted great attention in the field of artificial intelligence, and convolutional neural network (CNN) [4,5] performs well in recognition. A large amount of research on radar emitter signal recognition has been carried out using CNN. Qu et al. [6] trained a CNN model and deep Q-learning network, which use time-frequency images extracted by Cohen class time-frequency distribution as the input. Shao et al. [7] proposed a deep fusion method based on CNN, which provides competitive results in terms of classification accuracy. Wang et al. [8] combined the time-frequency maps and instantaneous autocorrelation maps of radar signals and used the joint feature maps as the input of CNN, which overcomes the weakness of a single feature map for the classification. Liu et al. [9] proposed an algorithm of radar emitter signal recognition, which uses the time-frequency images as the input of CNN. Cain et al. [10] combined radar frequency, pulse width and pulse repetition interval and used CNN for individual radar identification. Xiao et al. [11] proposed a method based on CNN, which uses the frequency features of automatic dependent surveillance broadcast (ADS-B) signal. Akyon et al. [12] classify the intra-pulse modulation of radar signals based on feature fusion and CNN.

However, in the field of radar emitter signal recognition, most of the sampled radar signals are one-dimensional (1-D) time-domain sequences. If we use the original two-dimensional (2-D) CNN models directly, it will take more time and storage space to transfer the sequences from 1-D form to 2-D form. Moreover, the dimensional transformation will result in poor real-time performance when the 2-D CNN models are used in practical applications. Although CNN models focus on global information and are able to extract features, the weights of the features are not the same, which means that the redundant and useless features can make recognition accuracy suppressed. Considering these limitations, this paper proposes a novel one-dimensional convolutional neural network with an attention mechanism (CNN-1D-AM) to extract features directly from original radar signals sequence in the time domain and focus on the key information of extracted features for radar emitter signal recognition.

The contribution of this paper can be concluded as follows:

(1) The 1-D convolutional layers can directly extract the feature from the time-domain sequences of radar signals. Moreover, compared with 2-D structure, 1-D convolutional layers save time in the dimensional transformation of radar signals, which makes the model better real-time performance in practical applications.

(2) A unit that employs an attention mechanism [13,14] is added to automatically weight the feature maps given by 1-D convolutional layers so that the important features can obtain more weights and the features which have negative impacts on recognition can be inhibited. The experimental results show that the proposed CNN-1D-AM can achieve high accuracy and has superior performance in radar emitter signal recognition.

This paper is organized as follows: In Section 2, the proposed CNN-1D-AM, which uses 1-D convolution and an attention mechanism, is introduced in detail. The experiments and discussions of the proposed methods and other compared methods are shown in Section 3. The conclusion is presented in Section 4.

## 2. One-Dimensional Convolutional Neural Network with Attention Mechanism (CNN-1D-AM)

### 2.1. One-Dimensional Convolution

CNN are usually designed to process 2-D data, especially images. As radar emitter signals are mainly in 1-D form and dimensional transformation is time-consuming, this paper proposed 1-D convolutional layers for feature extraction. The 1-D convolutional layers decrease the number of parameters compared with traditional 2-D convolutional layers. Moreover, the 1-D signals in the time domain are no longer converted into 2-D feature maps, which saves time and storage space.

Given the 1-D signal sequences ${\left\{x_{i}\right\}}_{i=1}^{N}$ where $x_{i}$ is the $i_{th}$ sample and N is the number of sequences. Assume that there are K filters in the first 1-D convolutional layer and L is the length of one signal sequence, which is the same as the input shape of the layer. Then the output of the filter in 1-D convolutional layer can be written as follows:

(1) $$y_{i}^{k}=f\left(w^{k}*x_{i}+b^{k}\right)$$

where $y_{i}^{k}$ denotes the output of the $k_{th}$ filter, f(⋅) is the activation function, $w^{k}$ and $b^{k}$ are the weight and bias of the $k_{th}$ filter, and ‘∗’ means convolution computation. When padding the edge of output result with zero, the output of 1-D convolutional layer can be written as $Y\in R^{L\times K}$.

Similar to 2-D CNN, a pooling layer is connected after the convolutional layers in 1-D CNN. The output of 1-D pooling layer can be written as $\tilde{Y}\in R^{\frac{L}{r}\times K}$, where r is the rate of downsampling. A typical structure of CNN can be written as follows:

(2) $$\begin{array}{cc} x_{i} & \to Y^{1}\to {\tilde{Y}}^{1}\to Y^{2}\to \\ {\tilde{Y}}^{2} & \to \ldots \to Y^{i}\to {\tilde{Y}}^{i}\to \ldots \end{array}$$

where $Y^{i}$ denotes the output matrix of the $i_{th}$ convolutional layer and ${\tilde{Y}}^{i}$ is the output matrix of the $i_{th}$ pooling layer.

### 2.2. Attention Unit

**Figure 1:**
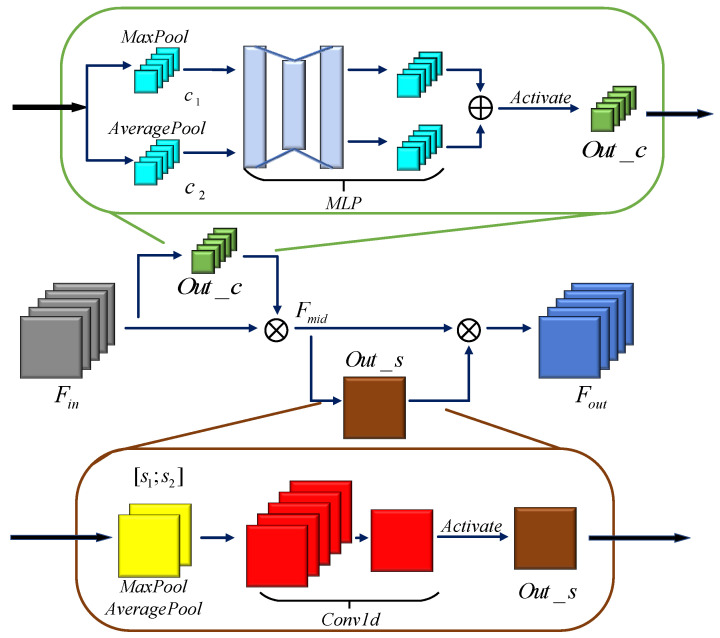
The structure of a one-dimensional attention unit (AU-1D).

In recent years, Woo et al. [15] proposed the convolutional block attention module (CBAM) in a 2-D CNN. CBAM has proven that the order of the channel attention first and the spatial attention later performs better. This paper proposes the one-dimensional attention unit (AU-1D), which is similar to the order of the original CBAM. The AU-1D is added between the last pooling layer and the first full connection layer, where the unit helps to capture the essential features and suppress the less important information. The structure of the proposed AU-1D is shown in Figure 1.

Given a feature map $F_{in}\in R^{W\times C}$, where W is the length of the map, and C is the number of channels. AU-1D first extracts the channel features by two ways of pooling. The max-pooling function and average-pooling function in the channel domain can be written as follows:

(3) $$c_{1}=MaxPool\left(F_{in}\right)\;=\max(F_{in}\left(1\leq i\leq W,C\right))$$

(4) $$c_{2}=AveragePool\left(F_{in}\right)\;=\;\frac{1}{W}\overset{W}{\underset{i}{\sum }}F_{in}\left(i,C\right)$$

where $c_{1}\in R^{1\times C}$ and $c_{2}\in R^{1\times C}$ are two different vectors calculated by different ways of pooling. Then, a multilayer perceptron (MLP) is used to extract features from $c_{1}$ and $c_{2}$ further. By activating the vector which is merged by two output feature vectors from MLP, the map of channel attention $Out\_c\in R^{1\times C}$ is produced. This process is shown as follows:

(5) $$Out\_c=Activate\left(MLP\left(c_{1}\right)+MLP\left(c_{2}\right)\right)$$

The map of channel attention can be considered as a feature detector [16]. It refers to the weight for each channel in the feature map. Different convolutional kernels extract different information in the channel domain. The map of channel attention refers to the weight of each channel. The more useful information the channel brings, the more weight the channel obtains.

Then, the middle-regained feature map $F_{mid}$ is obtained through the process of multiplying Out_c and the original feature map $F_{in}$. This process is shown as follows:

(6) $$F_{mid}=F_{in}⊗Out\_c\;=F_{in}⊗\sigma \left(W_{MLP}\left(c_{1}\right)+W_{MLP}\left(c_{2}\right)\right)$$

where ⊗ stands for multiply computation, σ denotes the sigmoid function, $W_{MLP}$ denotes the weights of MLP.

In spatial feature extraction, there are two ways of pooling whose pooling-axes [17] are different from that in channel feature extraction. The max-pooling function and average-pooling function in the spatial domain can be written as follows:

(7) $$s_{1}=MaxPool\left(F_{mid}\right)\;=\max(F_{mid}\left(W,1\leq j\leq C\right))$$

(8) $$s_{2}=AveragePool\left(F_{mid}\right)\;=\;\frac{1}{C}\overset{C}{\underset{j}{\sum }}F_{mid}\left(W,j\right)$$

where $s_{1}\in R^{W\times 1}$ and $s_{2}\in R^{W\times 1}$ are two different vectors calculated by different ways of pooling. $s_{1}$ and $s_{2}$ are concatenated into a fusion vector $s\in R^{W\times 2}$. The Conv1d unit extracted information from s. By activating the output of the Conv1d unit, the map of spatial attention $Out\_s\in R^{W\times 2}$ is produced. This process is shown as follows:

(9) $$s=[s_{1};s_{2}]$$

(10) Out_s=Activate(conv1d(s))

where conv1d(⋅) is the computation of 1-D convolution.

The map of spatial attention reflects the importance of features in different areas. Not all areas in the feature map are equally important to the recognition, but the areas which are relevant to the task of recognition should be concerned more.

Finally, the regained feature map $F_{out}$ is obtained through the process of multiplying Out_s and the original feature map $F_{mid}$. This process is written as follows:

(11) $$F_{out}=F_{mid}⊗Out\_s\;=F_{mid}⊗\sigma (W_{conv1d}\left(\left[s_{1};s_{2}]\right)\right)$$

where $W_{conv1d}$ denotes the weights of convolutional layers.

Through the AU-1D, the feature maps extracted from the 1-D convolutional layers will be weighted. The most useful information in the feature maps weights higher, and the useless information will be suppressed. In this way, the network can extract more effective features and improve the performance of recognition.

### 2.3. CNN-1D-AM

**Figure 2:**
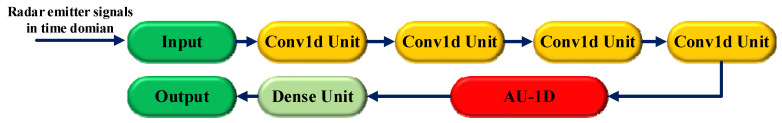
The structure of one-dimensional convolutional neural network with an attention mechanism (CNN-1D-AM).

According to the analysis of the 1-D convolution and attention unit, the structure of the CNN-1D model with attention mechanism (CNN-1D-AM), this paper proposed is shown in Figure 2.

In Figure 2, ‘Input’ is the layer, which uses the sequence of radar emitter signals in the time domain. ‘Output’ is the layer with a certain number of neurons, which refers to the number of signal types. ‘Conv1d Unit’ contains one convolutional layer, one max-pooling layer and one batch-normalization layer. The size of the convolutional kernels is 33 in four ‘Conv1d Units,’ and the number of filters is 32, 64, 128, 256 in turns. ‘Dense Unit’ contains one full connection layer.

To reduce the influence of different amplitudes on recognition, the amplitude normalization for the original data is needed. The original data are the radar emitter signals in the time domain. The expression of amplitude normalization is shown as follows:

(12) $$d\left(i,j\right)=\frac{r\left(i,1\leq j\leq H\right)}{max(abs\left(r\left(i,1\leq j\leq H\right)\right))},1\leq i\leq N$$

where $r\in R^{N\times H}$ are the original data sequences in the time domain, $d\in R^{N\times H}$ are the normalized data sequences in the time domain, N is the number of samples, and H is the length of each sample. The result of amplitude normalization is the input of the CNN-1D-AM model for recognition.

The activation function in the last layer is the ‘SoftMax’ function so that the probability for each type of signal in recognition can be obtained. The final probability for each type of signals is shown as follows:

(13) $${\hat{y}}^{i}=P(y=i|out)=\frac{e^{out^{i}}}{\sum_{i=1}^{T}e^{out^{i}}}$$

where $\hat{y}=\left[{\hat{y}}^{1},{\hat{y}}^{2},\ldots ,{\hat{y}}^{T}\right]$, $out^{i}=\left[out^{1},out^{2},\ldots ,out^{T}\right]$. ${\hat{y}}^{i}$ refers to the probability that the input data are recognized as class i. $out^{i}$ is the output of the $i_{th}$ neuron in the final output layer, which contains T neurons in total. The category corresponding to the maximum $\hat{y}$ is the classification result of CNN-1D-AM.

The cross-entropy (CE) function is selected as the cost function. The CE function is written as follows:

(14) $$L\left(\theta \right)=-\overset{T}{\underset{i=1}{\sum }}y^{i}\ln({\hat{y}}^{i})\;=-\overset{T}{\underset{i=1}{\sum }}y^{i}\ln(g\left(\theta ,x{)}^{i}\right)$$

where y is the one-hot coded result of data label, g(θ,x) denotes the output of CNN-1D-AM with x as the input, θ is the weights of the model, L(θ) is the result of the CE function.

Adaptive moment estimation (ADAM) [18] is chosen as the optimization algorithm. According to (14), this algorithm can be written as follows:

(15) $$g\leftarrow \nabla_{\theta }L\left(\theta \right)$$

(16) $$m\leftarrow \beta_{1}m+\left(1-\beta_{1}\right)g$$

(17) $$v\leftarrow \beta_{2}v+\left(1-\beta_{2}\right)g^{2}$$

(18) $$m\leftarrow m/\left(1-\beta_{1}^{T}\right)$$

(19) $$\theta \leftarrow \theta -\alpha \cdot m/\left(\sqrt{v}+\varepsilon \right)$$

where g is the gradient of L(θ) by its gradient operator $\nabla_{\theta }$, m and v are the moment vectors with 0 as their initial value, $\beta_{1}$ and $\beta_{2}$ are constants, usually set to 0.9 and 0.999, α is the learning rate, ε is a smoothing parameter, typically set to $10^{-8}$.

## 3. Experiments and Discussions

The experiment platform parameters for algorithm implementation are shown in Table 1.

### 3.1. Dataset

Seven different varieties of radar emitter signals were used to validate the effectiveness of the proposed algorithm, namely, continuous wave (CW), linear frequency wave (LFM), nonlinear frequency wave (NLFM), binary phase-shift keying (BPSK), quadrature phase-shift keying (QPSK), binary frequency shift keying (BFSK) and quadrature frequency shift keying (QFSK). These seven different types of modulation are commonly used in radar systems. The specific parameters of the signals are shown in Table 2. The carrier frequency and frequency bandwidth change within a certain range, which meets the changing characteristics of the electromagnetic environment.

The datasets in the experiment were produced like this:

(1) First, we generated seven types of radar emitter signals with different values of SNR. The type of noise was Gaussian white noise, and the passband ranged from 90 MHz to 340 MHz. The SNR for each type of signal ranged from −10 dB to 0 dB with 1 dB step, totaling 11 values. The number of samples for each type of signal with each value of SNR was 7000.

(2) Second, we divided the samples into three different datasets. As (1) shows, 7000 samples for each type of signal with each value of SNR were divided into training dataset with 1600 samples, validation dataset with 400 samples and testing dataset with 5000 samples.

(3) Third, we made the final datasets. The final training dataset with 123,200 samples, the final validation dataset with 30,800 samples and the final testing dataset with 385,000 samples were combined by the datasets in (2).

### 3.2. Experiments of CNN-1D-AM

The model CNN-1D-AM was trained based on the preprocessed data in Section 3.1. The number of parameters and training time per epoch for CNN-1D-AM is shown in Table 3.

As shown in Table 3, the training time of CNN-1D-AM for each epoch with 123,200 samples was less than one minute, which means that the model was lightly designed and was on low incremental resource consumption.

**Figure 3:**
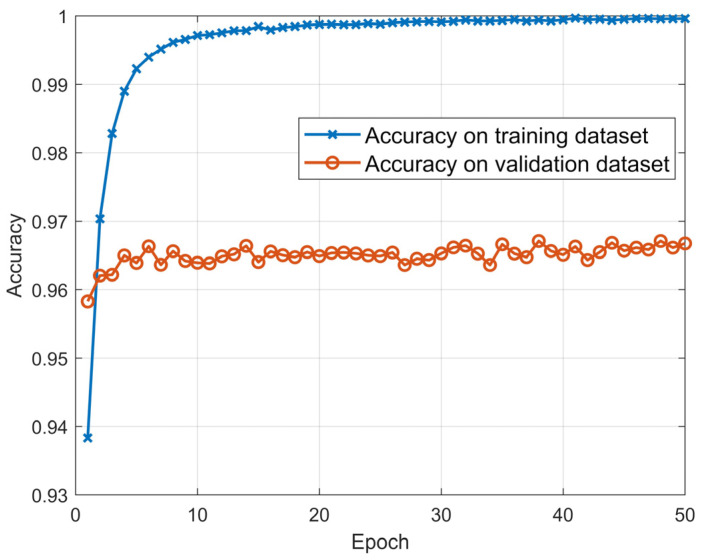
The average recognition rates of CNN-1D-AM on the training dataset and validation dataset with different quantity of training epochs.

The average recognition rates for the training dataset and validation dataset during the training session are shown in Figure 3.

Figure 3 shows that after training 50 epochs, the recognition accuracy of CNN-1D-AM on the training dataset reached nearly 100%. Moreover, the recognition accuracy of the model on the validation dataset was over 96%, which denotes that the model converged.

**Figure 4:**
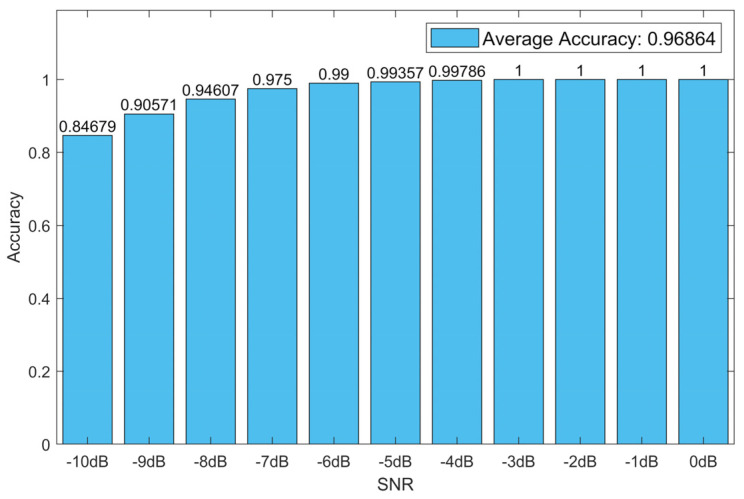
The recognition rates of CNN-1D-AM with 11 values of signal-to-noise ratio (SNR) on the validation dataset.

The weights of the neural network with the highest recognition rate on the validation dataset were saved. Under this circumstance, the recognition rate of CNN-1D-AM with 11 values of SNR on the validation dataset is shown in Figure 4.

Figure 4 indicates that the model acquired nearly 100% accuracy when the SNR was above −6 dB. Moreover, the accuracy was less than 90% only when SNR was lower than −9 dB.

**Figure 5:**
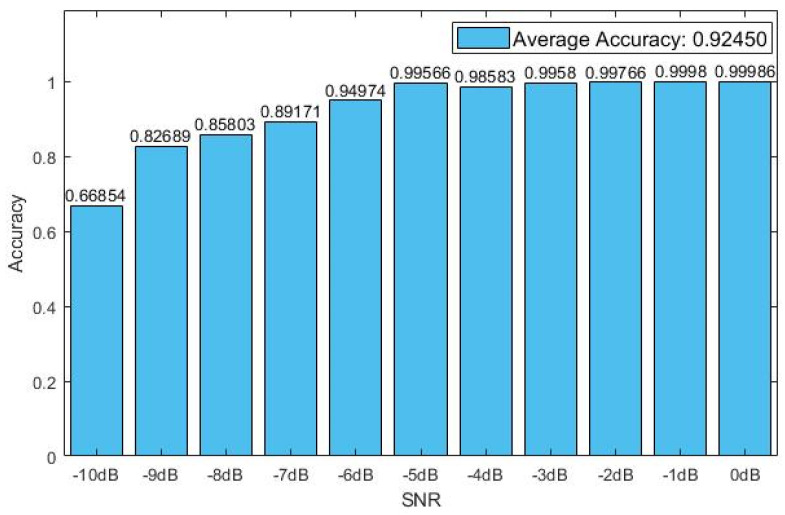
The recognition rates of CNN-1D-AM with 11 values of SNR on the testing dataset.

In the real applications, the number of samples which need to be tested is always larger than that on the validation dataset. Therefore, the testing dataset with large-scale samples was used to validate the exact real performance of the model. The recognition rate of CNN-1D-AM with 11 values of SNR on the testing dataset is shown in Figure 5.

As shown in Figure 5, the average recognition rate of CNN-1D-AM decreased compared with Figure 4. This is because the number of samples on the testing dataset was about 12.5 times more than that on the validation dataset and 3.125 times more than that on the training dataset. This is equivalent to the situation that a model is trained with fewer samples and is tested with a huge number of samples. When SNR was above −5 dB, the accuracy of recognition on the testing dataset was still close to 100%. Interestingly, the recognition rate fell nearly 1% when the SNR rose from −5 dB to −4 dB.

**Figure 6:**
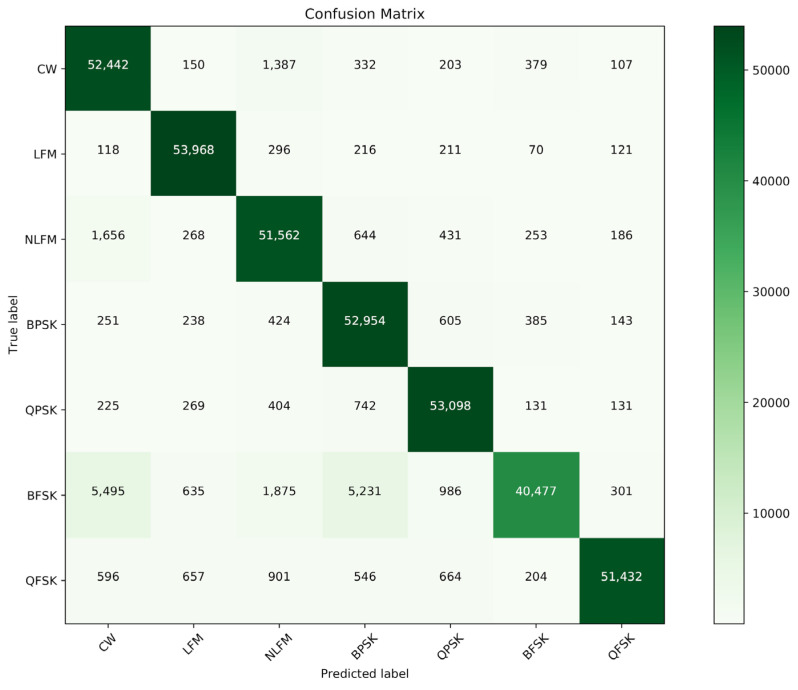
The confusion matrices of CNN-1D-AM, based on average recognition rates.

To figure out the specific recognition results of CNN-1D-AM, the confusion matrix for average recognition performance based on the testing dataset is shown in Figure 6. It was found that the part of low recognition rates could be attributed to the classification of BFSK signals. A portion of the BFSK signals was mainly misidentified as CW signals and BPSK signals. Apart from this, the average recognition rates of the other six types of signals were over 93.5% by calculating.

### 3.3. Learned Features

**Figure 7:**
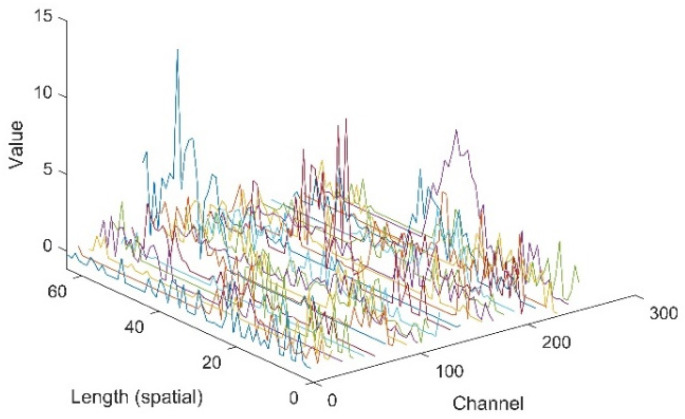
The features filtered by the layer before the attention unit.

**Figure 8:**
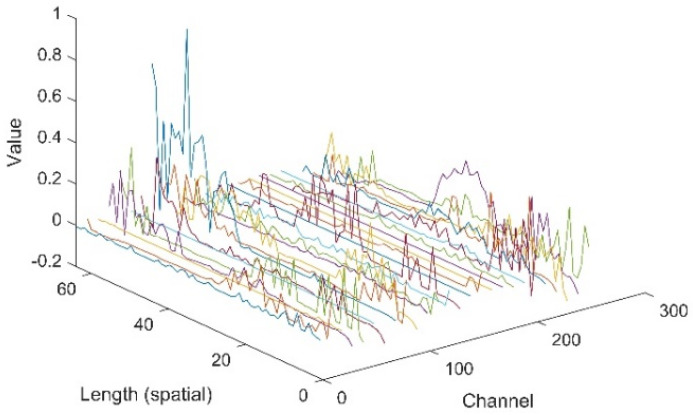
The features weighted by the attention unit.

**Figure 9:**
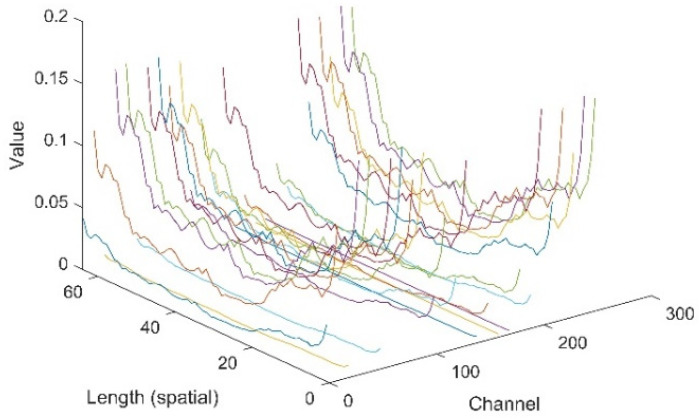
The weights of the attention unit.

In this section, the extracted features of signals by the proposed CNN-1D-AM were investigated. Specifically, a sample from the testing dataset was sent to the CNN-1D-AM model. Some features filtered by the layer before the attention unit and weighted by the attention unit are plotted in Figure 7 and Figure 8. The weights of the attention unit are also shown in Figure 9.

Figure 7 and Figure 8 indicate that the features in different channels and different positions of space were weighted by the attention unit. The relative values of features in some certain channels and in some positions of space turned to zero. Moreover, Figure 9 shows that the features in different positions of space and channels gained weights differently based on the attention unit.

### 3.4. Comparison of Other Methods

To further evaluate the effectiveness of the proposed method, some traditional methods and state-of-the-art deep learning-based models were used as a comparison.

The traditional methods include SVM [19], which uses seven HOC features as the input; SSAE1, which uses spectral power feature, amplitude feature in the time domain and six HOC features as input. Moreover, the deep learning-based models include CNN and deep neural networks (DNN) [20], stacked autoencoder (SAE) [21].

For the CNN part, the VGG network [22] and ResNet [23] were chosen as the comparison models. As the structure of the proposed CNN-1D-AM is not complicated, for this paper, we chose the specific VGG network, which includes 13 weight layers (VGG13) and the specific ResNet, which includes 18 layers (ResNet18). To make the comparison between methods as fair as possible, both of VGG13 and ResNet18 were transferred from 2-D forms, and the parameters were reset properly according to the literature. Moreover, to investigate the impact of the attention mechanism, a CNN-1D model, which is transferred by deleting the attention unit from the proposed models, was also used as a comparison (CNN-1D-Normal).

For the DNN part, four different models were chosen, and the detail of these models is shown in Table 4. The adjacent layers were fully connected. The differences among the four DNN models were the quantity of layers and the number of neurons in the layers.

In addition, three SAE models were chosen, and their structure is shown in Table 5. The SAE models included at least one autoencoder and one classifier. Moreover, the adjacent layers of autoencoders and the classifier were fully connected.

The datasets used in this session were the same as before. The input of CNN, DNN and SAE models in comparison was the sequences of radar emitter signals in the time domain. Moreover, the input data of SVM and SSAE were calculated according to the same datasets.

**Figure 10:**
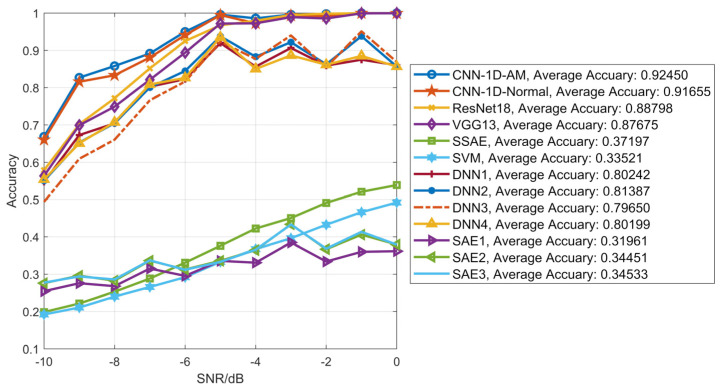
Recognition accuracy of different methods and models (CNN-1D-AM, CNN-1D-Normal, ResNet18, VGG13, SSAE, SVM, DNN1, DNN2, DNN3, DNN4, SAE1, SAE2, SAE3) with each value of SNR on the testing dataset.

Figure 10 shows the recognition accuracy of different methods and models with each value of SNR on the testing dataset. By analysis, the accuracy of convolutional neural network models was higher than other methods, and the performance of CNN-1D-AM this paper proposed was superior to those of other models above-mentioned. Moreover, the comparison between CNN-1D-AM and CNN-1D-Normal shows that AU-1D could improve the recognition accuracy of the network.

Table 6 shows the number of parameters and training time per epoch for convolutional neural network models, which indicated that the CNN-1D-AM model was of higher efficiency and lower consumption of computation.

## 4. Conclusions

This paper proposes a novel CNN-1D-AM for radar emitter signal recognition. The designed 1-D convolutional layers especially could directly extract features from the time-domain sequences of radar emitter signals. The attention unit was integrated into the CNN-1D model so that the recognition accuracy of a neural network could be improved further. The experimental results indicated that CNN-1D-AM could achieve high accuracy of recognition on seven different radar signals. The comparison results with some traditional methods and deep learning-based models show the superior performance of CNN-1D-AM. In future work, we hope to propose a CNN-1D model with a new attention mechanism, which can increase the accuracy of recognition further.

## Figures and Tables

**Table 1:** Experiment platform parameters.

Project	Parameter
CPU	Intel Silver 4110
GPU	P400 + P40
RAM	64 GB
System Version	Centos 7
Simulation Software	MATLAB2020a, Python3.7, Keras 2.2.4

**Table 2:** Specific parameters of seven types of radar emitter signals.

Signal Type	Carrier Frequency	Parameter
CW	200 MHz~220 MHz	None
LFM	200 MHz~220 MHz	Frequency bandwidth: 50 MHz to 60 MHz
NLFM	200 MHz~220 MHz	Frequency of modulation signal ranges from 10 MHz to 12 MHz
BPSK	200 MHz~220 MHz	13-bit Barker code Width of each symbol is 0.038 us
QPSK	200 MHz~220 MHz	16-bit Frank code Width of each symbol is 0.03 us
BFSK	200 MHz~220 MHz 300 MHz~320 MHz	13-bit Barker code Width of each symbol is 0.038 us
QFSK	100 MHz~110 MHz 150 MHz~160 MHz 200 MHz~210 MHz 250 MHz~260 MHz	16-bit Frank code Width of each symbol is 0.03 us

Note 1: The pulse width for each type signal is 0.5 us; Note 2: Sampling frequency is 2 GHz.

**Table 3:** Quantity of parameters and training time per epoch for CNN-1D-AM.

Model	CNN-1D-AM
Quantity of parameters	3,554,504
Time per epoch	55 s

**Table 4:** The detail of four deep neural networks (DNN) models for radar emitter signal recognition.

Neurons of the Layers	DNN1	DNN2	DNN3	DNN4
Input layer	1024			
First hidden layer	512	512	256	512
Second hidden layer	256	256	64	256
Third hidden layer	128	N/A	N/A	128
Fourth hidden layer	N/A	N/A	N/A	64
Output layer	7			

**Table 5:** The structure of the stacked autoencoder (SAE) model for radar emitter signal recognition.

SAE Model	Parts of SAE	First Auto-Encoder	Second Auto-Encoder	Third Auto-Encoder	Classifier
SAE1	Input layer	1024	512	256	128
	Hidden layer	512	256	128	N/A
	Output layer	1024	512	256	7
SAE2	Input layer	1024	512	N/A	256
	Hidden layer	512	256		N/A
	Output layer	1024	512		7
SAE1	Input layer	1024	N/A		512
	Hidden layer	512			N/A
	Output layer	1024			7

**Table 6:** The number of parameters and training time per epoch for convolutional neural network models.

Model	CNN-1D-AM	CNN-1D-Normal	ResNet18	VGG13
Quantity of parameters	3,554,504	3,520,903	4,465,543	5,761,863
Time per epoch	55 s	50 s	101 s	80 s
